# Human papillomavirus genotyping and integration in ovarian cancer Saudi patients

**DOI:** 10.1186/1743-422X-10-343

**Published:** 2013-11-20

**Authors:** Othman A Al-Shabanah, Mohamed M Hafez, Zeinab K Hassan, Mohamed M Sayed-Ahmed, Waleed N Abozeed, Salem S Al-Rejaie, Abdulmalik A Alsheikh

**Affiliations:** 1 Department of pharmacology, College of pharmacy; King Saud University, P.O. Box 2457, Riyadh 11451, Kingdom of Saudi Arabia; 2 Zoology department, Faculty of science, King Saud University, Riyadh, Kingdom of Saudi Arabia; 3 Medical Oncology Unit; King Khalid University Hospital; King Saud University, Riyadh, Kingdom of Saudi Arabia; 4 Pathology department, College of Medicine, King Saud University, Riyadh, Kingdom of Saudi Arabia

**Keywords:** DNA sequencing, Formalin paraffin embedded tissue, Human papilloma virus, Ovarian cancer, Polymerase chain reaction

## Abstract

**Background:**

Human papillomavirus (HPV) is associated with different malignancies but its role in the pathogenesis of ovarian cancer is controversial. This study investigated the prevalence, genotyping and physical state of HPV in ovarian cancer Saudi patients.

**Methods:**

Hundred formalin fixed paraffin embedded (FFPE) ovarian carcinoma tissues and their normal adjacent tissues (NAT) were included in the study. HPV was detected by nested polymerase chain reaction (PCR) using degenerated HPVL1 consensus primer pairs MY09/MY11 and GP5+/GP6 + to amplify a broad spectrum of HPV genotypes in a single reaction. The HPV positive samples were further genotyped using DNA sequencing. The physical state of the virus was identified using Amplification of Papillomavirus Oncogene Transcripts (APOT) assay in the samples positive for HPV16 and/or HPV18.

**Results:**

High percentage of HPV (42%) was observed in ovarian carcinoma compared to 8% in the NAT. The high-risk HPV types 16, 18 and 45 were highly associated with the advanced stages of tumor, while low-risk types 6 and 11 were present in NAT. In malignant tissues, HPV-16 was the most predominant genotype followed by HPV-18 and -45. The percentage of viral integration into the host genome was significantly high (61.1%) compared to 38.9% episomal in HPV positive tumors tissues. In HPV18 genotype the percentage of viral integration was 54.5% compared to 45.5% episomal.

**Conclusion:**

The high risk HPV genotypes in ovarian cancer may indicate its role in ovarian carcinogenesis. The HPV vaccination is highly recommended to reduce this type of cancer.

## Introduction

Human papillomavirus (HPV) belongs to Papillomaviridae family that consists of small double stranded DNA viruses associated with cutaneous and mucosal squamous epithelial lesions [1]. HPV infection is detected in cancers of the female lower genital tract [2,3]. However, its role in the development of cancers in the upper genital tract, such as endometrial and ovarian cancer, is less clear [4]. More than 200 genotypes of HPV have been identified and were subdivided into two groups the oncogenic and non-oncogenic group [5]. The oncogenic HPV genotypes are 16, 18, 31, 33, 35, 39, 45, 51, 52, 56, 58, 59, 66 and 68 [6,7]. Of these, type 16 and 18 have been classified as “high-risk” (HR-HPV) because they are associated with the malignant progression of cervical tumors and with other genital and head-neck malignancies [8]. The high-risk HPV types produced two oncogenes, designed E6 and E7 proteins induce transformation by interference with endogenous cell cycle regulatory proteins, including P53, retinoblastoma (Rb) and breast cancer type 1 susceptibility protein (BRAC1) [9]. The L1 open reading frame (ORF) region is the most conserved gene within the HPV genome, and has been used for identification of genotyping and new HPV genotypes [10]. The HPV detection in clinical samples is based on the DNA fragments amplification in the L1 region [11].

Ovarian carcinoma is the most lethal gynecological malignancy because it is detected in advanced stages with 5-year survival rate is <40% [12,13]. The etiology of ovarian cancer remains unclear and may be multifactorial. Ovarian cancer is either epithelial carcinomas or malignant germ cell tumors [14]. Its incidence rates are the highest among developed countries, with rates exceeding 9/100,000 women per year. In Saudi Arabia, ovarian cancer is the seventh most common malignancy among females and accounting for 3.1% of all newly diagnosed cases with median age of 50 years [15]. Epithelial ovarian cancer accounts for 85-90% of total ovarian tumor [16].

The genetic alterations associated with ovarian carcinomas are well known [17,18]. The risk factors that lead to ovarian carcinomas include positive family history of ovarian, breast or colon cancer; old age; number of ovulations; endocrine factors; endometriosis; pelvic inflammation; fat intake [19]. Other environmental factors as HPV infection are recently under investigations. The participation of HPV infection could be suspected to be involved in the development of ovarian cancer [20]. Several studies provided highly controversial results [21-26].

The integration site of HPV is extensively investigated in cell lines and clinical samples of HPV related cancers at various sites of the body. The integration site for HPV is random throughout the genome but the Integration mechanisms are not fully understood [27]. Integration of HPV into host cell genome is found with a high percentage in cervical cancer infected with HPV16 and HPV18 genotypes, and low in the precancerous lesions and undetected in early HPV-induced lesion [28-30]. In late stage of cancers, viral integration into host genome is important in the disease progression. Viral integration occurs downstream of the early genes E6 and E7 or in the E1 or E2 region causing gene inactivation [31]. Therefore, the integration of viral genomes may contribute to a large extent to the neoplastic transformation process and may trigger chromosomal instability. There are number of methods for the detection of HR-HPV integrants in human genome such as Amplification of Papillomavirus Oncogene Transcripts (APOT), Restriction Site PCR (RS-PCR), Southern blot and Detection of Integrated Papillomavirus Sequences (DIPS). The APOT method is able to detect the integration of viral genome in clinical lesions even in the presence of a large excess of non-integrated, episomal, form of viral genomes [32]. The current study aimed to determine the prevalence, genotyping and physical state of HPV in cancerous and normal adjacent tissues from ovarian cancer Saudi patients.

## Results

**Figure 1:**
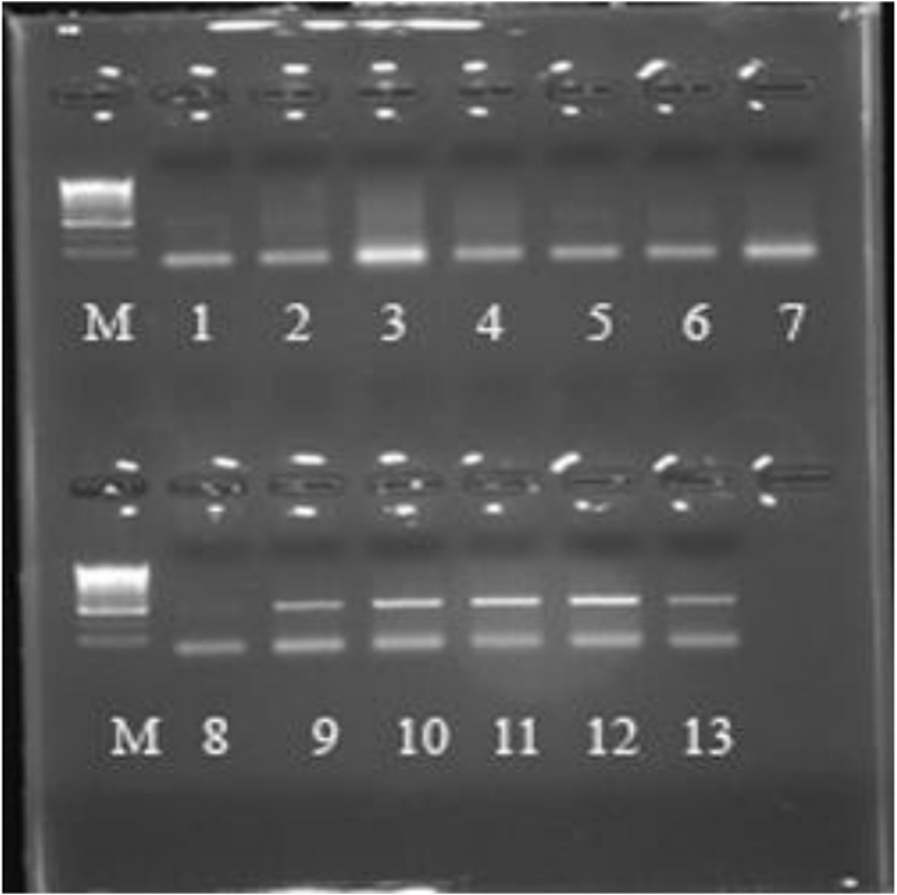
**PCR products were analyzed on a 2% agarose gel stained with ethidium bromide and visualized by UV-trans-illumination.** Lane M is 50 PCR marker (Promega), Lanes 1, 5 and 8 are weak positive sample, lanes 2, 3, 4, 6 and 7 are negative samples, from lane 9-12 are positive samples and lane 13 is positive control.

Genomic DNA was isolated from 100 FFPE ovarian carcinoma and their normal adjacent tissues. All samples were positive for β-globin gene amplification. The amplified HPV DNA with MY09/MY11 followed by GP5+/GP6+ were considered HPV positive and were subjected to DNA sequencing. The negative samples by nested PCR were subjected to HPV-type specific PCR to confirm that the samples were negative. Forty two out of 100 tumor samples and 8 out of 100 NAT were positive for HPV by nested PCR as shown in Figure 1.

The patients’ mean age was 50 ± 11 years (range, 25–78 years). The patients’ age distribution was analyzed and the prevalence of HPV genotypes was detected among them. There was no significant difference (P > 0.5) observed in HPV infection among patients with age <45 compared to >45 years old, in which 15/35 (42.8%) of the patients with age <45 years old and 27/65 (41.5%) were positive for HPV. The HPV infection in relation to histological grade, HPV was detected in 25% (7/28) of cases with grade I, 50% (21/42) of cases with grade II and 46.7% (14/30) of cases with grade III.

**Figure 2:**
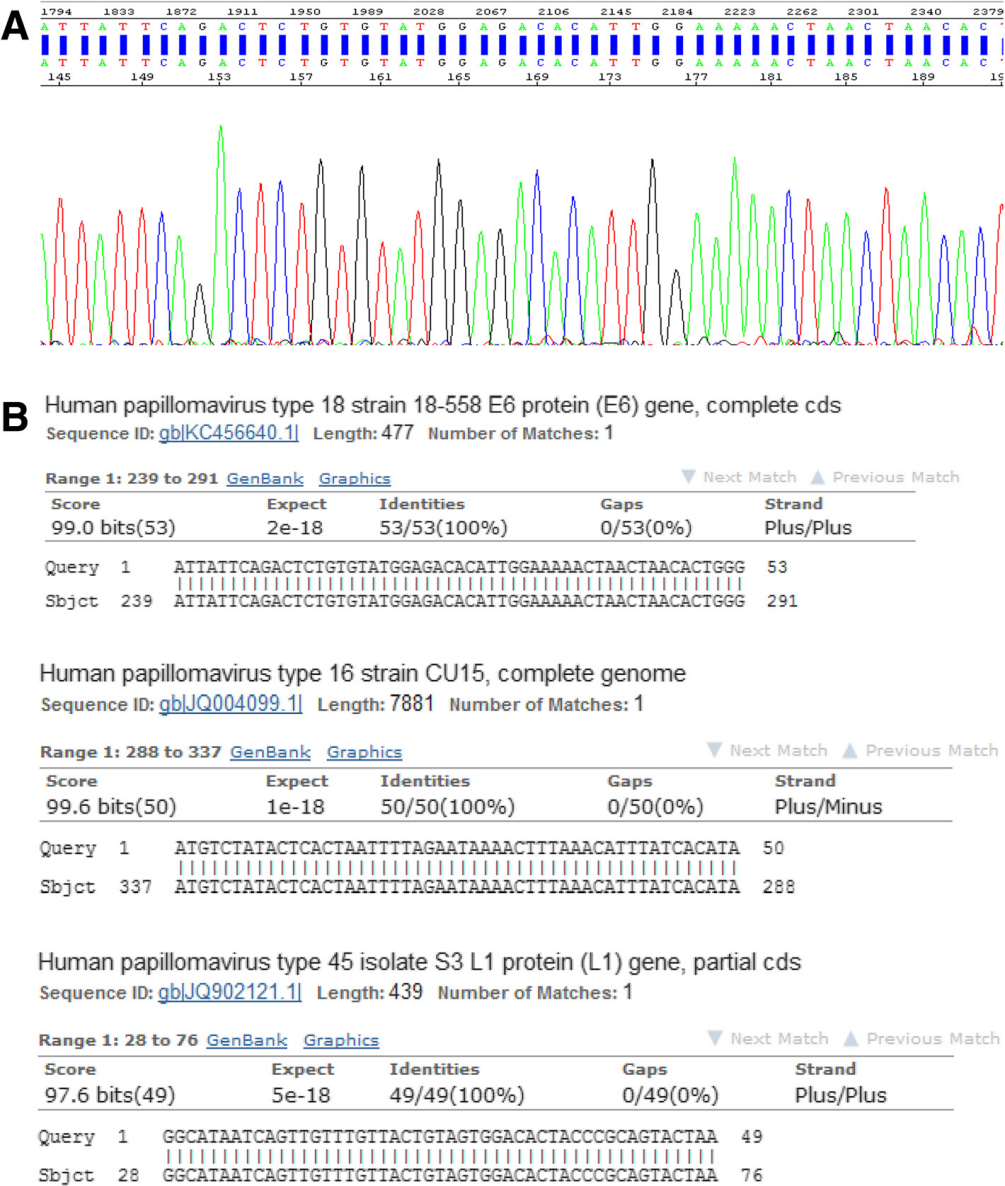
Sequencing data of HPV genotypes (A) A sequence excised from an electropherogram for HPV type 18 (B) Sequence alignment of HPV type 16, 18 and 45 using Basic Local Alignment Search (BLAST).

By using sequencing technique, the most common detected HPV genotype was HPV-16 in 18/42 (42.9%), followed by HPV-18 in 11/42 (26.2%), finally HPV-45 in 7.1% as in Figures 2A and 2B. The overlapped sequences was seen in 10/42 (23.8%) cases that indicates the presences of more than one HPV genotype.

As the sequencing technique failed to identify the specific mixed HPV genotypes, therefore type specific PCR assay was used. Mixed infection with HPV-16/18, HPV-16/45, HPV-18/45 or HPV = 16/18/45 were observed in 7/42 (16.6%), 2.4%, 2.4%, 2.4% respectively as showed in Table 1. The prevalence of HPV-6 and HPV-11 (low risk HPV genotypes) were detected only in NAT HPV positive samples with 50% of both genotypes (Table 1).

**Table 1:** The prevalence of different human papillomavirus genotypes in ovarian cancer and its adjacent normal tissues

**HPV infection**	**Ovarian cancer**	**Normal adjacent tumor**
Total positive	(42/100) 42%	(8/100) 8%
HPV-16	(18/42) 42.9%	0 (0%)
HPV-18	(11/42) 26.2%	0 (0%)
HPV-16/18	(7/42) 16.6%	0 (0%)
HPV-45	3/42 (7.1%)	0 (0%)
HPV-16/45	1/42 (2.4%)	0 (0%)
HPV-18/45	1/42 (2.4%)	0 (0%)
HPV-16/18/45	1/42 (2.4%)	0 (%)
HPV-6	0 (0%)	4/8 (50%)
HPV-11	0 (0%)	4/8 (50%)

**Figure 3:**
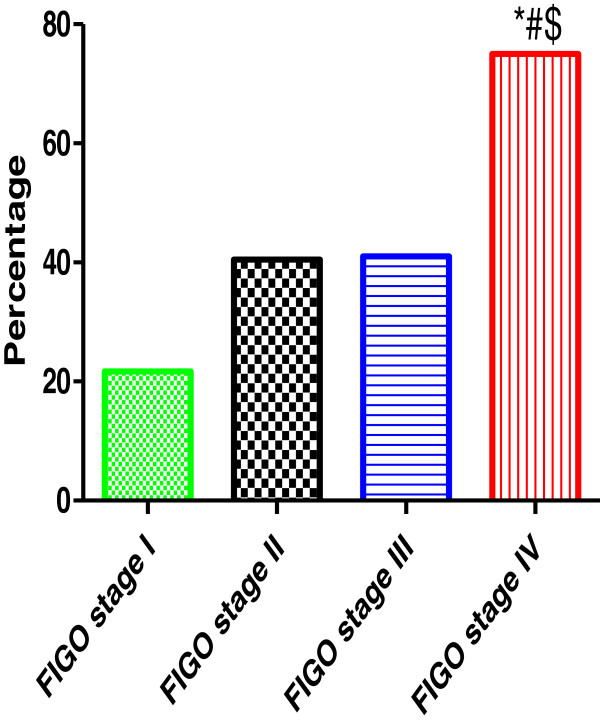
**The incidence of human papillomavirus in relation to ovarian cancer stages.** *,# and $ indicate significant difference from Stage I, stage II and stage III respectively.

The clinical stages were determined according to the International Federation of Gynecology and Obstetrics system (FIGO). The ovarian cancer samples were classified as followed: stage 1-23%, stage II-37%, stage III-29% and stage IV-11%. HPV was detected in 21.7% (5/23) stage 1, 40.5% (15/37) stage II, 41% (16/39) stage III and 75% (9/11) stage IV as showed in Figure 3. The presence of HPV infection was significantly higher in patients with advanced stages (FIGO stage III and IV) 50% compared to localized disease (FIGO stage I and II) 33.3% (P < 0.05).

**Figure 4:**
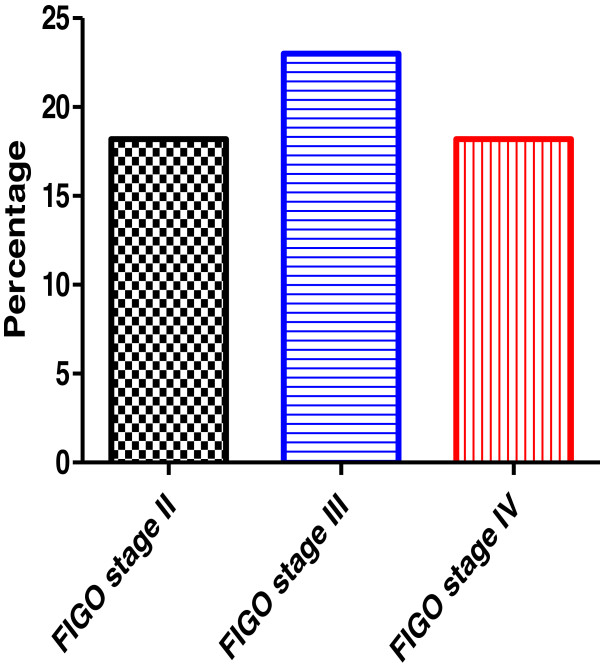
The incidence of human papillo mavirus type 16 in relation to ovarian cancer stages.

**Figure 5:**
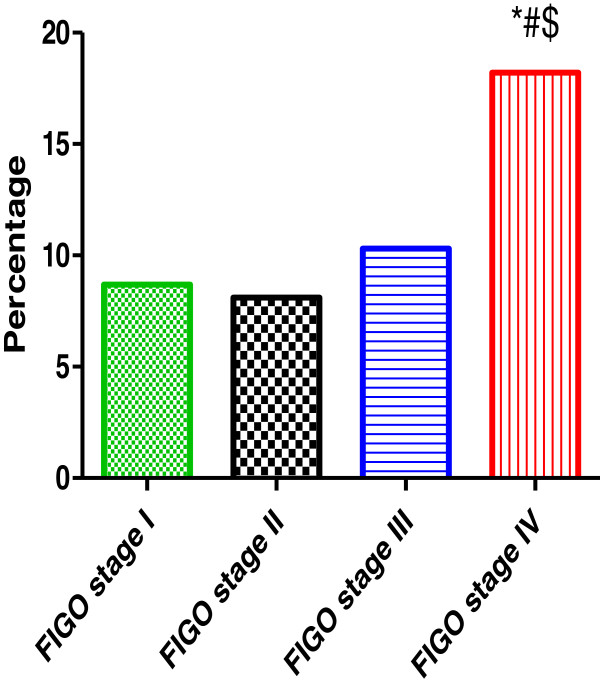
**The incidence of human papillomavirus type 18 in relation to ovarian cancer stages.** *, # and $ indicate significant difference from stage I, satge II and stage III, respectively.

The HPV genotypes were distributed among different FIGO stages as followed: HPV-16 were (7/37) 18.9%, (9/39) 23% and (2/11) 18.2% in stage II, III and IV respectively as showed in Figure 4. HPV-18 was 2/23 (8.7%) in stage I, 3/37 (8.1%) in stage II, 4/39 (10.%) in stage III and 2/11 (18.2%) in stage IV (Figure 5). On the other hand HPV-45 was observed in 2 cases ofstageI and in one case with stage II.

The APOT assay is based on the structural differences among the 3′-ends of viral oncogene transcripts. The integration of HR-HPV genomes into the host genome results in both disruption of the *E1* or *E2* open reading frames and deletion of viral early-region from the viral oncogene-encoding sequences. Thirty six HPV-16, HPV-18 or HPV-16/-18 positive samples with good quality RNA, were subjected to study the physical states by APOT assay. The viral genome was found to be integrated in 22/36 (61.1%), whereas the episomal transcripts were found in 38.9%. Out of the 22 cases with integrated viral genome the episomal form of HPV was also detected in 5/22 (22.7%). In HPV-18 genotype positive samples the percentage of viral integration was 54.5% compared to 45.5% episomal. On the other hand in HPV-16 genotype positive samples the percentage of viral integration was 65% compared to 35% episomal. The integrated state of the virus was significantly found in 90% in advanced stages III and IV compared to 10% in localized stages I and II (p < 0.05) showing that the integrated form was associated with the advanced stages of cancer.

## Discussion

The role of HPV infection in cervical cancer [33] and other types of cancer [34-37] has been studied. The role of HPV in ovarian cancer development is debated [24,38-43] may be due to the different samples size or the technique used to detect HPV. Therefore, subsequent studies are in need to confirm the potential impact of HPV in ovarian cancer. In Saudi Arabia, ovarian cancer represents the seventh most common malignancy and cancer-related deaths among females [15]. To the best of our knowledge, this is the first study on the association between HPV infection and ovarian cancer in Saudi Arabia. This study investigated the presence of HPV genotypes and its physical states in ovarian cancer Saudi women.

HPVs are classified as high and low risk, according to their relationship with benign or malignant proliferative lesions [44]. The oncogenic activity of high-risk HPV types occurred when they integrated in the host genome.

In many studies, molecular assays were used to identify different types of HPV in cells and tissues [45-47]. The use of MY09/MY11 followed by GP5+/GP6+ primers to enhance the detection sensitivity in samples containing low viral copy numbers and to amplify a wide spectrum of HPV genotypes [48]. In the present study, the MY09/MY11 and GP5+/GP6+ primers followed by DNA sequencing and type specific PCR were used to confirm the HPV genotypes and to identify the mixed infection. In the current study, the incidence of HPV was higher in cancerous tissues than in NAT by both the PCR and sequencing techniques. Some studies detect the high incidence of high-risk HPV DNA in both benign and ovarian malignant tumors [49,50]. Similar study indicates the importance of HPV in malignant development via the statistical difference of HPV distribution in benign compared to malignant ovarian tissue of Chinese patients [4]. In contrast, other study didn’t find any association between ovarian cancers and the presence of HPV infection by using PCR assay [51]. A study in India included 20 ovarian cancer biopsies, demonstrated complete absence of HPV infection in ovarian cancer [52]. Other studies have shown lower rate of HPV DNA in ovarian than cervical cancer [4,24]. Hence, the identification of HPV DNA in ovarian tumors may provide an evidence of a metastatic cervical cancer [53,54]. The geographical variation in HPV variants showed that the virus and the host has co-evolved over time [55]. In our study, the identification of high-risk HPV in cancerous tissues reflects the HPV possible role in ovarian carcinogenesis and the viral type is probably an important factor. Some host genetic factors is implicated in HPV persistence such as polymorphism or variation is human major histocompatibility class II [56]. Other study on the cervical cancer Saudi patients found high incidence of HPV with HPV-16 and -18 the common genotypes. They concluded that the HPV prevalence in cervical cancer patients is comparable to the international rates [8].

In the current study, the high-risk HPV types 16, 18 and 45 were associated with advanced stages of the disease, while low-risk types 6 and 11 were associated with normal tissues adjacent to the tumor. This finding was in agreement with other study found that the presence of high-risk types of HPV was found in the serous histological subtype and advanced stages of the disease (FIGO stages III and IV) [39]. Other studies showed no evidence of high-risk HPV association with histological subtype and/or stage of disease [4,24]. In ovarian carcinoma Serbia patients, HPV infections were more frequent in FIGO III/IV in relation to FIGO I/II stages [57].

There is no statistical significant difference in HPV infection in relation to age group more/or less than 45 years. The current study are consistent with those reports based the detection of HPV in relation to age, they found that the median age of diagnosis of ovarian cancer patients infected with HPV was 57 years for patients with HPV and 59 years for patients without HPV infection [4].

HR-HPV DNA integration in cervical cancer genome plays an important role in cancer pathogenesis [1]. HPV-transformed cells growth rate depends on *E6* and *E7* oncogenes expression level [58,59], so the integration is important in HR-HPV-induced cancer. Therefore, the viral genomes integration resulted in elevated expression levels of the *E6* and *E7* oncogenes [60].

Integration of HPV is common in late stage cervical cancers and considered as an important event in disease progression. The molecular detection of integrated HR-HPV genomes may represent a suitable marker for the identification of invasive carcinoma. Integration generally occurs in E1 or E2 region downstream of the early genes E6 and E7. Viral E2 gene is well known to play a role in viral replication as well as negative regulation of E6 and E7 genes [39] and transcriptionally inactivated once the virus gets integrated due to disruption of its open reading frame. Some studies showed that both episomal and integrated forms are able to transform normal keratinocytes [61-63].

The APOT assay allows distinguishing between integrate- and episome-derived transcripts encompassing HR-HPV E7 sequences. To study the physical state of HPV, APOT assay was used to detect the integration of viral genome [32]. In the current study, the percentage of viral integration into the host genome was detected in 61.1% of HPV positive tumors and was 38.9% episomal. The incidence of integration in HPV18 positive samples was 54.5% compared to 45.% the virus was episomal. Also the virus integrated state was associated with the advanced stages of cancer. This was inconsistent with other study that was done on the relationship between integration of HPV and cervical cancer [32]. Similar study found, by applying APOT assay to samples infected with HR-HPV types 16 and 18, a strong correlation between the detection of integrate-derived transcripts and the progression stage of the cervical dysplasia. Some investigators detected integrated HPV genomes in various preneoplastic lesions [64] or in invasive carcinoma samples [65]. This discrepancy was attributed to the different methodological approaches used for the detection of integrated HPV DNA [65].

## Conclusion

This study supports the hypothesis that there was a correlation between HPV infection and ovarian cancer in Saudi Arabia. The high percentage of HR-HPV associated with ovarian cancer and its integrated form may reflect a possible role of this virus in the carcinogenesis of ovarian tumors or it may facilitate its progression. From this study, we recommended theadmission of HPV vaccination in the national vaccination program.

## Material and methods

Human papillomavirus and its integration were investigated in ovarian cancer and its normal adjacent tissues(NAT). The study was conducted in compliance with Helsinki Declaration and was approved by the review board of King Khalid Hospital, King Saud University. It included 100 archival formalin-fixed paraffin embedded (FFPE) ovarian cancer and their normal adjacent tissues were collected from Pathology Department, College of Medicine, King Saud University and Riyadh Regional Laboratory and blood bank. All the pathological data were taken from the pathological reports. The mean age were 50 ±11 years ranging from 25–78 years.

### Nucleic acid extraction

Cervical cancers cell lines positive for HPV-16 (SiHa, CaSki) and HPV-18 (HeLa, C-4 I) were used as positive control for genotypes and for viral integration. All FFPE samples were thin sectioned at 8 μm thicknesses using Leica Microtome (Manual Rotary Microtome RM2235). Tissue sections were floated in a DEPC-treated water bath then picked up on glass slides then allowed to dry.

Genomic DNA was used for HPV genotyping and RNA was used to detect the integration of HPV. Three tissue sections were used for RNA and DNA extraction using Recover All total Nucleic Acid Isolation Kit (Ambion, Life Technologies, USA) following the manufacturer instructions. In brief the tissue sections were deparaffinized then digested by proteases. The nucleic acid was isolated by preparing the isolation additive/ethanol mixture followed by transfer to the column then eluted. The quantity and quality of the RNA and DNA were characterized using a UV spectrophotometer (NanoDrop8000, Thermo scientific, USA).

### Human papillomavirus detection by nested PCR

The specimens were amplified with beta-globin primers GH20 and GH21 in order to check the DNA quality. Nested PCR with consensus primers MY09/MY11 was used to amplify a wide spectrum of HPV types with PCR product of 450 bp followed by GP5+/GP6+ with PCR product of approximately 150 bp as previously described [66]. Each sample was tested three times. The primers sequences are shown in Table 2. The PCR reaction was done in 50 μl, contained 500 ng of DNA, 1XPCR Master Mix (Promega, Madison, USA), 3 mMMgCl2, 300 nM of each primer. Amplifications using MY09/MY11were performed with the following cycling profile: incubation at 94°C for 5 min followed by 40 cycles of 1 min denaturation at 95°C, 1 min annealing at 55°C, and 1 min elongation at 72°C. The last cycle was followed by a final extension of 10 min at 72°C. The annealing step of GP5+/GP6+ primers-based PCR was performed at 40°C for 2 min. During amplification positive and negative control samples were included. PCR products were analyzed on a 2% agarose gel stained with ethidium bromide and visualized by UV-transillumination Figure 1.

**Table 2:** Primers sequence used in this study

**Primers**	**Forward primer**	**Reverse primer**
**MY09/MY11**	**5′-CGTCC(AC)A(AG)(AG)GGA(T)ACTGATC-3′**	**5′-GC(AC)CAGGG(AT)CATAA(CT)AATGG-3′**
**GP5+/GP6+**	**5′-TTTGTTACTGTGGTAGATACTAC-3′**	**5′-GAAAAATAAACTGTAAATCATATTC-3′**
**HPV-16**	**5′-CAC AGT TAT GCA CAG AGC TGC-3′**	**5′-CAT ATA TTC ATG CAA TGT AGG TGTA-3′**
**HPV-18**	**5′-CAC TTC ACT GCA AGA CAT AGA-3′**	**5′-GTT GTG AAA TCG TCG TTT TTC A-3′**
**HPV-31**	**5′-GAA ATT GCA TGA ACT AAG CTC G-3′**	**5′-CAC ATA TAC CTT TGT TTG TCA A-3′**
**HPV-59**	**5′-CAA AGG GGA ACT GCA AGA AAG-3′**	**5′-TAT AAC AGC GTA TCA GCA GC-3′**
**HPV-45**	**5′-GTG GAA AAG TGC ATT ACA GG-3′**	**5′-ACC TCT GTG CGT TCC AAT GT-3′**
**HPV-33**	**5′-ACT ATA CAC AAC ATT GAA CTA-3′**	**5′-GTT TTT ACA CGT CAC AGT GCA-3′**
**HPV 6/11**	**5′-TGC AAG AAT GCA CTG ACC AC-3′**	**5′-TGC ATG TTG TCC AGC AGT GT-3′**
**HPV-58**	**5′-GTA AAG TGT GCT TAC GAT TGC-3′**	**5′-GTT GTT ACA GGT TAC ACT TGT-3′**
**HPV-52**	**5′-TAA GGC TGC AGT GTG TGC AG-3′**	**5′-CTA ATA GTT ATT TCA CTT AAT GGT-3′**
**HPV-56**	**5′-GTG TGC AGA GTA TGT TTA TTG-3′**	**5′-TTT CTG TCA CAA TGC AAT TGC-3′**
**HPV-35**	**5′-CAA CGA GGT AGA AGA AAG CAT C-3′**	**5′-CCG ACC TGT CCA CCG TCC ACCG-3′**
**HPV-42**	**5′-CCC AAA GTA GTG GTC CCA GTT A-3′**	**5′-GAT CTT TCG TAG TGT CGC AGT G-3′**
**HPV-43**	**5′-GCA TAA TGT CTG CAC GTA GCT G-3′**	**5′-CAT GAA ACT GTA GAC AGG CCA AG-3′**
**HPV-44**	**5′-TAA ACA GTT ATA TGT AGT GTA CCG-3′**	**5′-TAT CAG CAC GTC CAG AAT TGA C-3′**
**HPV-68**	**5′-GCA GAA GGC AAC TAC AAC GG-3′**	**5′-GTT TAC TGG TCC AGC AGT GG-3′**
**HPV-39**	**5′-GAC GAC CAC TAC AGC AAA CC-3′**	**5′-TTA TGA AAT CTT CGT TTG CT-3′**
**HPV-51**	**5′-GAG TAT AGA CGT TAT AGC AGG-3′**	**5′-TTT CGT TAC GTT GTC GTG TAC G-3′**
**HPV-66**	**5′-TTC AGT GTA TGG GGC AAC AT-3′**	**5′-AAA CAT GAC CCG GTC CAT GC-3′**
**GAPDH**	**5′-CCACTCCTCCACCTTTGA-3′**	**5′-ACCCTGTTGCTGTAGCCA-3′**
**GH20 and GH21**	**5′-GAA GAG CCA AGG ACA GGT AC-3′**	**5′-CAA CTT CAT CCA CGT TCA CC-3′**
**P1-16**	**5′-CGGACAGAGCCCATTACAAT-3′**	
**P1-18**	**5′-TAGAAAGCTCAGCAGACGACC-3′**	
**P3**	**5′-GACTCGAGTCGACATCG-3′**	
**P2-16**	**5′-CTTTTTGTTGCAAGTGTGACTCTACG-3′**	
**P2-18**	**5′-ACGACCTTCGAGCATTCCAGCAG-3′**	
**(dt)17-P3-**	**5′GACTCGAGTCGACATCGATTTTTTTTTTTTTTTTT-3′**	

### Type‒specific PCR

Multiple infections and negative samples were subjected to type-specific PCR to confirm the results. The amplification reactions were performed using 18 HPV primers as previously described [67] (13 for HR-HPV 16, 18, 31, 33,45, 35, 39, 51, 52, 56, 58, 66, and 68 and 5 for Low risk HPV 6,11,42,43 and 44) in separate reactions. Each reaction was performed in a final volume of 50 μL containing 500 ng of DNA, 1 × PCR Buffer 300 nM of each primer, and 1 U of Taq polymerase (KAPABIOSYSTEM,USA). The amplification conditions were 95°C for 10 min followed by 40 cycle of 1 min denaturation at 95°C, 1 min annealing temperature vary for each primers, and 2 min extension at 72°C. The last cycle was followed by a final extension of 10 min at 72°C.

### Amplification of papillomavirus oncogene transcripts (APOT)

cDNA was synthesized from 1 *μ*g of total RNA by reverse transcription using an oligo(dT)17-primer coupled to a linker sequence p3 using a high capacity cDNA kit (Applied biosystem, life technology, USA), according to the manufacturer’s instructions. To control RNA integrity and cDNA quality, PCR reactions using glyceraldehyde-3-phosphate dehydrogenase–specific primers were performed.

cDNAs include viral oncogene sequences were subsequently amplified by PCR using HPVE7–specific primer [P1-16] for HPV16 and [P1-18] for HPV18 as forward primers and linker p3 as the reverse primer. The PCR was performed in 50 μL reaction volume containing 5 μL of the RT reaction mixture (cDNA), 2.5 units Taq polymerase (promega madison wisconsin, USA), 1× PCR buffer (500 mMKCl, 1.5 mM MgCl2), 200 μM each of the deoxyribonucleotide triphosphate and 0.25 μM of each primer. The reaction mixture was subjected to initial denaturation for 2 min, followed by 35 cycles of denaturation at 94°C for 45 s, annealing at 58°C for 45 s, elongation at 72°C for 2 min, and a final elongation step at 72°C for 7 min. Five μl of the amplified products were used as template for nested PCR under the same conditions at annealing temperature 65°C using forward HPVE7-specific primer [p2-16] specific for HPV16 and [P2-18] specific for HPV18 as forward primers and (dT)17-p3 as reverse primer [64].

### DNA sequencing

To identify the HPV genotypes and the integration, all positive PCR products were analyzed by direct DNA sequencing. PCR products were purified using QIAquick Purification Kit according to manufacturer’s instructions (QIAGEN, Hilden, Germany). Purified PCR products were labeled with fluorescent dyes using BigDye Terminator v3.1 Cycle Sequencing Kit Applied Biosystem. Labeled oligonucleotides were purified using BigDye X Terminator Purification Kit (Applied Biosystems, CA, USA).

The samples were sequenced by automatic ABI 3500 genetic analyzer (Applied Biosystems, USA). Chromatograms with sharp peaks and quality values ≥20 with little or no background noise consider as single HPV infection. When the samples contain more than one HPV genotype, direct sequencing gave mixed chromatograms, with overlapping peaks or two or more fluorescent signals. Samples with mixed chromatogram were subsequently subjected to type-specific PCR. The nucleotide sequences were subsequently subjected to Basic Local Alignment Search (BLAST) provided by the National Cancer Institute, USA.

### Statistics

Chi-square test used to test prevalence differences in HPV genotypes and integration frequencies between different HPV types in cancerous tissues and NAT in relation to age, tumor stage and tumor grade. A p value of < 0.05 was considered statistically significant. SPSS, version 17.0 was used for these analyses.

## Competing interests

The authors declare that they have no competing interests.

## Authors’ contributions

MMH and ZK shared in molecular studies and their statistical analysis and draft the manuscript. MMH, OA, MS, WN, SM and AA participated in study design, sample and data collection and shared in revising the manuscript. SS shared in sample and data collections. All authors read and approved the final manuscript.
